# Characterising Soil Eukaryotic Diversity From NEON Metagenomics Datasets

**DOI:** 10.1111/1755-0998.70062

**Published:** 2025-10-22

**Authors:** Leena Vilonen, Andrew Thompson, Byron Adams, Edward Ayres, André L. C. Franco, Diana H. Wall

**Affiliations:** ^1^ School of Global Environmental Sustainability, Colorado State University Fort Collins Colorado USA; ^2^ Kravis Department of Integrated Sciences Claremont Mckenna College Claremont California USA; ^3^ Ronin Institute Riverside California USA; ^4^ Department of Biology Brigham Young University Provo Utah USA; ^5^ National Ecological Observatory Network, Battelle Boulder Colorado USA; ^6^ Paul H. O'Neill School of Public and Environmental Affairs Indiana University Bloomington Indiana USA

**Keywords:** 18S, metagenomics, NEON, soil eukaryotes

## Abstract

Belowground eukaryotic diversity serves a vital role in soil ecosystem functioning, yet the composition, structure, and macroecology of these communities are significantly under‐characterized. The National Ecological Observatory Network (NEON) provides publicly available datasets from long‐term surveillance of numerous taxa and ecosystem properties. However, this dataset is not routinely evaluated for its eukaryotic component, likely because analyzing metagenomes for eukaryotic sequences is hampered by low relative sequence abundance, large genomes, poorer eukaryote representation in public reference databases, and is not yet mainstream. We mined the NEON soil metagenome datasets for 18S rRNA sequences using a custom‐built pipeline and produced a preliminary assessment of biodiversity trends in North American soil eukaryotes. We extracted ~800 18S rRNA reads per sample (~22,000 reads per site) from 1455 samples from 495 plots across 45 NEON sites in 11 biomes, which corresponded to 5183 genera in 35 phyla. To our knowledge, this represents the first large‐scale soil eukaryote analysis of NEON data. We asked whether taxonomic richness paralleled patterns previously established ecological trends and found that eukaryotic richness was negatively correlated with pH, managed sites lowered eukaryotic richness by 47%, most biomes had a distinct eukaryotic community, and fire decreased eukaryotic richness. These findings parallel generally accepted ecological trends and support the notion that NEON soil metagenome datasets can and should be used to explore spatiotemporal patterns in soil eukaryote diversity, its association with ecosystem functioning, and its response to environmental changes in North America.

## 1 Introduction

Belowground eukaryotic diversity is integral to ecosystem functioning worldwide (Delgado‐Baquerizo et al. 2020), yet the composition, structure, and macroecology of these communities are significantly under‐characterised (Geisen et al. 2018; Oliverio et al. 2020). This knowledge gap persists in part due to the high morphological and taxonomic diversity of soil eukaryotes (comprising soil animals, phagotrophic and phototrophic protists, and fungi), the complexity of soil communities (Anthony et al. 2023), the challenges of working with microscopic organisms embedded in a spatially complex and recalcitrant matrix (Flemming et al. 2023), and the complexity of eukaryotic genomes and life histories (del Campo et al. 2014). Nevertheless, the significance of belowground fauna to the maintenance of Earth's biosphere cannot be overstated: soils contain around one quarter of global animal biodiversity (Decaëns et al. 2006), which consume half of global leaf litter production annually (Heděnec et al. 2022), accelerate litter decomposition rates by 37% globally (Garcia Palacios et al. 2013), and significantly increase N and P availability to plants (Gebremikael et al. 2016). Not surprisingly, numerous studies report positive associations between soil faunal richness and ecosystem functioning (Delgado‐Baquerizo et al. 2020; Jing et al. 2015; Kou et al. 2021).

However, despite the growing availability of high‐throughput datasets, the molecular ecology of soil eukaryotes is still underexplored. The National Ecological Observatory Network (NEON) provides a well‐organised hierarchy of multi‐dimensional datasets (e.g., soil and water‐extracted metagenomes, above‐ and belowground abiotic variables such as, soil moisture, temperature, and solar irradiation, and abundance data on meso‐ and macroscopic organisms, such as, beetles and birds), including soil shotgun metagenomes from across the continental United States plus Alaska, Hawaii, and Puerto Rico that spans nearly a decade of sampling. NEON datasets are routinely used to evaluate trends in soil prokaryotes (Masuda et al. 2024; Chuckran et al. 2024), but to our knowledge, little to no work has been done on their eukaryotic component.

A central challenge in characterising soil fauna macroecology is the specialised taxonomic knowledge and intensive labor required for traditional morphological identification. High throughput amplicon and shotgun metagenomic sequencing provide an alternative to traditional morphological identification for studies with large sample sizes and broad taxonomic groups of interest. The application of these techniques in soil microbiology, for example, has facilitated profiling of soil microbial populations by circumventing limitations in extraction and culturing, and by streamlining access to the taxonomic expertise required to accurately characterise such communities (Guo et al. 2016). Similarly, shotgun metagenomics targeting soil invertebrate communities has been shown to accurately reflect taxonomy and reference genome properties (Schmidt et al. 2022). However, analysing eukaryotes with these techniques is more challenging and less developed than for prokaryotes (Lara et al. 2022; Bazant et al. 2023). Amplicon sequencing using universal primer pairs misses substantial micro‐eukaryotic biodiversity (Geisen et al. 2015), and shotgun metagenomes from complex environments are expensive to sequence deeply enough to recover sufficient eukaryote gene markers, which are often swamped by the high relative abundance of prokaryotic sequences (Guo et al. 2016). Though several tools have been developed for better recovery and identification of eukaryote sequences from shotgun metagenomes (e.g., Metaxa2, Eukdetect, Tiara, and Metaphlan6; Bengtsson‐Palme et al. 2015; Lind and Pollard 2011; Karlicki et al. 2022; Blanco‐Míguez et al. 2023), they rely on custom reference databases that are influenced by what is available in NCBI and SILVA, which often misrepresent the diversity of many eukaryotic lineages (Mugnai et al. 2023; Chorlton 2024). Curated databases focused on representing microeukaryotes more comprehensively do exist, such as the protist ribosomal database (PR^2^; Guillo et al. 2013), and thus a synthetic approach utilising diverse software and databases can help to overcome some of these challenges.

To better understand soil eukaryote diversity in North America and exploit a previously underused resource for exploring eukaryote diversity, we extracted, identified, and analyzed eukaryotic SSU rDNA sequences from shotgun metagenome datasets collected by NEON (1455 samples collected from 495 plots from 45 sites in 11 biomes throughout the US) using a custom pipeline capable of (1) handling data formats specific to NEON and (2) incorporating pre‐existing software specializing in the processing and analysis of eukaryotic sequences from shotgun metagenomics. Utilizing a eukaryote sensitive hmm profile and the curated protist ribosomal database (PR^2^), we extracted sequences aligning to the eukaryotic 18S rRNA gene and asked (1) whether there is sufficient eukaryotic sequence data in NEON shotgun metagenomes to conduct meaningful analyses; (2) if so, which are the most taxonomically rich eukaryotic phyla in US soils; and (3) as an initial validation of the dataset, whether the recovered patterns match generally accepted ecological trends. Specifically, we explore changes in soil eukaryotic biodiversity following fire and compare biodiversity at paired low‐ and high‐management intensity sites with the expectation that biodiversity would decrease following fire and be lower at more intensively managed sites. In addition, we explore biome‐level differences in community composition.

## 2 Methods

### 2.1 Soil Extraction, Library Preparation, Sequencing, and Data Management

**FIGURE 1:**
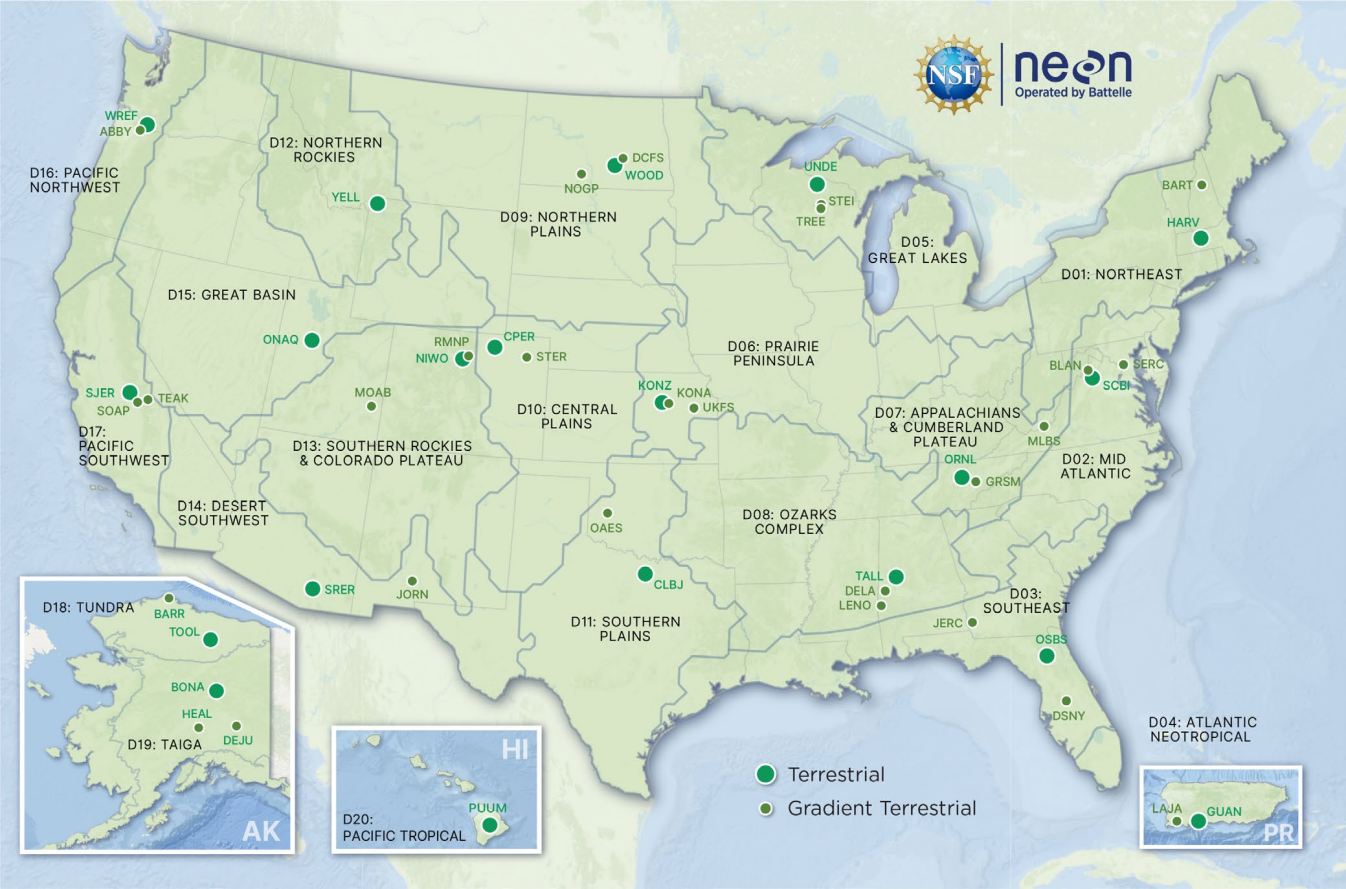
Locations of NEON core and gradient sites.

Soil extraction, library preparation, sequencing, and raw sequence data management were all performed by NEON following their standardized protocols (NEON 2022a). Soil samples are collected during the peak period of the growing season and initially collected annually at all sites but are currently collected annually at the 20 Core sites and every 5 years at the 27 Gradient sites (Figure 1). Samples are collected to a maximum depth of 30 cm (or restrictive feature if shallower), split into organic and mineral soil layers if an organic layer is present, then stored at −60°C to −85°C until they can be processed (NEON 2024). 136 site‐years of data from 45 of the 47 NEON terrestrial sites 12′ were available at the time of downloading and ~25 site‐years of new data are added annually (20 Core sites and ~5 Gradient sites). At each site, samples are collected from 10 plots distributed over 30 ± 20 km^2^ (median ± median absolute deviation) during each sampling event and the data used in this study come from 1455 samples collected from 495 plots across 45 sites. Plots span 11 biomes: evergreen forest, mixed forest, deciduous forest, woody wetland, shrub/scrub, dwarf scrub, grassland/herbaceous, sedge/herbaceous, pasture/hay, cultivated crops, and emergent herbaceous wetlands (NEON uses the US National Land Cover Database to classify vegetation type; NEON 2022b).

Whole genomic DNA was extracted from 0.25 ± 0.03 g of each thawed soil sample with the Qiagen DNeasy 96 PowerSoil Pro Kit (cat #47017), according to the manufacturer's instructions. The concentrations of extracted DNA were assessed using a Promega Quantus Fluorometer with a QuantiFluor ONE dsDNA Kit (#E4870) according to the manufacturer's instructions (Manual: Quantus_FluorometerManual_TM396_rev 01/2020). Shotgun metagenome libraries were made using the KAPA HyperPrep Kit from Kapa Biosystems, quantified using qPCR, normalized, then sequenced on an Illumina NextSeq 550 (Manual: 15069765v01) with read lengths of 2 × 150 bp and an insert length of 300 bp. Resulting sequences were uploaded to MG‐RAST for quality control, processing, and downstream analyses, then sent to NEON for storage in their public portal.

### 2.2 Bioinformatics

**FIGURE 2:**
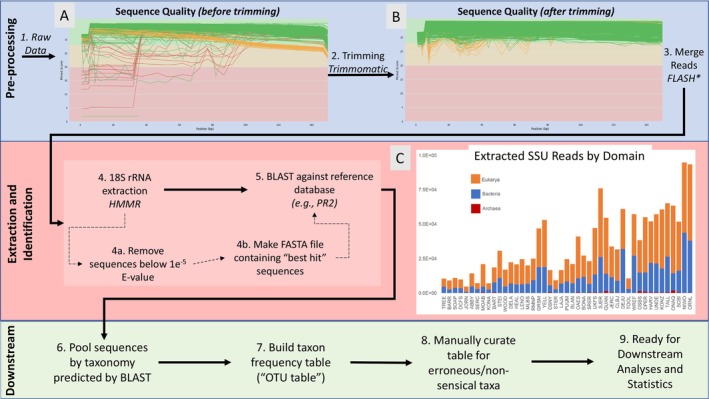
Pipeline flowchart with some sequence statistics. Pipeline step numbers in figure correspond to numbers in text. (A) phred scores for all bp positions across all raw sequences from all sites BEFORE trimming. Lower scores are represented by red lines in red areas of graph (i.e., < phred of 20). (B) phred scores for all bp positions across all raw sequences AFTER trimming. Note higher overall sequence quality. An estimated average 95.4% of sequences retained after trimming (data not shown). (C) Read abundance by domain by site (organised by number of sampling events per site, increasing to the right). Counts are of taxonomic ids made after HMMR extraction targeting 18S and do not represent relative abundances found in raw reads or nature. Bacterial and archaeal reads were likely retained due to shared conserved regions between 18S and 16S and are not likely artefacts. Curation for eukaryotic false positives were carried out in subsequent steps. Eukaryotic reads are broadly abundant (≥~1000) in every site.

To accommodate the changing tool landscape, continual data updates from NEON, and a need for customizable tool usage, we built an in‐house bioinformatics pipeline (Figure 2) that (1) handles data formats specific to NEON and (2) incorporates pre‐existing software and databases specially made for the processing and analysis of eukaryotic sequences from shotgun metagenomics. The present iteration of this pipeline was developed for use within our lab group only and currently is not guaranteed to be cross‐platform compatible (e.g., via dockerized containment), does not contain robust error‐catching, checkpoints, or thorough documentation, but future development could expand its functionality to include additional eukaryote‐specific tools. The latest version of the pipeline's source code can be found on GitHub (see data availability).

Our pipeline used an hmm profile to retrieve 18S sequences from shotgun metagenomes (Wheeler and Eddy 2013; Seemann 2018; see Figure 2), as done previously (Thompson et al. 2020) and in a way functionally analogous to the approach in Metaxa2 (Bengtsson‐Palme et al. 2015). Briefly, the pipeline (A) sorts then merges raw forward and reverse fastq files using fastq‐tools (Jones 2020) and FLASH (Magoč and Salzberg 2011), respectively (note that sorting was required as the sequence order in paired‐end files downloaded from NEON's online portal did not match). Merging is performed prior to trimming following Maran and Davis (2022), and the default parameters are used except for ‐M (max‐overlap), which was set to 150 bp. The pipeline next (A) evaluates the sequence quality of the merged raw data using FastQC, (2) trims low quality nucleotides or whole reads using Trimmomatic with the following settings: LEADING 2, TRAILING 2, SLIDINGWINDOW 4:15 (default), MINLEN 30 (Bolger et al. 2014), then (B) checks the post‐trimming sequence quality using FastQC (Andrews 2010) and MultiQC (Ewels et al. 2016). After trimming, reads matching the target marker gene (the 18S rRNA for this paper) are (3) extracted using nhmmer (Wheeler and Eddy 2013) with the eukaryote hmm profile developed for the rRNA prediction software Barrnap and an e‐value cutoff of 1e‐5 (Seemann 2018), filtered by hit score, and (4) aligned against the PR^2^ database, version 4.10.0 (Guillo et al. 2013), using BLASTn v2.7.1+ (Camacho et al. 2009). Plastid sequences, sequences shorter than 125 bp or with query coverage less than 90%, and sequences with an identity score below 93% are then removed (note that sequences undergo two trimming steps is an artefact of the pipeline's design to ultimately accommodate multiple approaches and to reduce downstream computation time in downstream analyses). Though such a conservative identity threshold could potentially bias against underrepresented microeukaryote clades (e.g., non‐metazoan and fungal kingdoms) and thus weaken the power of our study, our use of the PR^2^ database, which emphasizes the breadth of eukaryotic diversity, and the shortness of our reads (~138 bp) relative to the size of the full 18S gene (~2.5 kb) largely mitigates this risk. Unlike targeted metagenomics where PCR amplifies a specific region of a marker gene that is consistent across all individuals sampled, shotgun metagenome libraries provide random coverage of whole sequenced genomes. Given sufficient sequencing depth, these randomized pieces can be assembled (i.e., lengthened), then aligned for greater identification accuracy and precision. However, eukaryote sequences are generally represented in low abundance in soil shotgun metagenomes due to low relative abundance in environmental samples and DNA extraction biases (Santos et al. 2015) and can be difficult to assemble unless especially deep sequencing is performed (Commichaux et al. 2002). As the NEON extraction and sequencing protocols follow the standard procedures for prokaryotes (e.g., 0.25 g soil extracted) and no specific strategies were employed to ensure the capturing of eukaryotes, eukaryote sequence density was not high enough to perform assembly. To get around the limitations of using relatively short sequences to identify taxa against the 18S rRNA gene (Wu et al. 2015), the pipeline (5) groups extracted eukaryote sequences by their taxonomic assignment (i.e., in this case, genus) according to the taxonomy of our reference database (PR^2^). These assignments were used (6) to build “taxon frequency” tables and (7) manually checked for erroneous or nonsensical taxa (e.g., marine taxa) prior to downstream analyses. The latest version of the pipeline can be found on GitHub (Andy‐Thmpsn 2025—see data availability). To ground‐truth the approach used in the pipeline, we ran our data through Eukdetect on default settings.

### 2.3 Site Properties

Soil properties for each metagenomics sample were from (NEON 2022a). NEON site management data (NEON 2022b) was used to determine the CLBJ soil plots that burned between the metagenomics soil sampling in April 2017 and 2018. Properties for paired sites used to assess the impact of lower and higher management intensities on soil biodiversity are shown in Table 1. The paired sites span different regions of the US and cover a wide range of climates (e.g., mean annual temperature, MAT: 4°C–25°C; mean annual precipitation, MAP: 344–2451 mm), but each set of paired sites has similar climates (median difference in MAT and MAP is 0.7°C and 15 mm, respectively) and are relatively nearby (median distance between sites: 27 km). Since management type and intensity can vary within the site sampling boundary, the impact of management intensity was assessed based on data from the NEON tower base plots only (i.e., excluding distributed plots) because the tower plots are typically managed similarly within a site, whereas the distributed plots may encompass different management types.

**TABLE 1:** Site details for paired lower and higher management intensity sites.

Site ID	MAT^a^ (°C)	MAP^b^ (mm)	Dominant NLCD vegetation classes^c^	Management intensity (type)	Distance between site pairs (km)	Region
WREF	9.2	2225	EF	Lower	30	Washington
ABBY	10	2451	EF|GH|SS	Higher (forestry)		
UNDE	4.3	802	DF|MF|WW	Lower	81	Wisconsin/Michigan
STEI	4.8	797	DF|MF|WW	Higher (forestry)		
KONZ	12.4	870	DF|GH	Lower	4	Kansas
KONA	12.7	850	CC	Higher (cropland)		
CPER	8.6	344	GH	Lower	150	Colorado
STER	9.7	433	CC	Higher (cropland)		
WOOD	4.9	494	EHW|GH	Lower	11	North Dakota
DCFS	4.9	490	GH	Higher (cattle grazing)		
GUAN	23	840	EF	Lower	23	Puerto Rico
LAJA	25	830	CC|GH|PH	Higher (cattle grazing)		

^a^ Mean annual temperature.

^b^ Mean annual precipitation.

^c^ National Land Cover Database Vegetation classes: CC, Cultivated Crops; DF, Deciduous Forest; EF, Evergreen Forest; EHW, Emergent Herbaceous Wetlands; GH, Grassland/Herbaceous; MF, Mixed Forest; PH, Pasture/Hay; SS, Shrub/Scrub; WW, Woody Wetlands.

### 2.4 Data Analyses

Abundance counts for each site were normalised using a Hellinger transformation using the labdsv package for all analyses below. OTU tables were analysed in R version 4.41 with the mctoolsr package (Leff 2022) and vegan version 2.3‐5 (Oksanen et al. 2016). Rarefaction (Figure S1) and species accumulation curves (Figure S2) were generated to visualise sequencing depth while vegan was used to visualise taxon abundance. NEON site characteristics relevant to the study, such as, elevation, latitude, soil temperature, and soil moisture, were also included in the analysis (see Table 2 for all site characteristics). Mixed models using the lme4 and lmerTest packages were used to evaluate regressions between OTU richness and site characteristics, where the site characteristics were the fixed variable and site and plot were random effects. To evaluate trends in our data, our study used mixed model regressions for several site characteristics to assess whether relationships with OTU richness were present (seen in Table 2). Analyses focused on the major groups (fungi, metazoa, etc.) as well as the three most abundant metazoan phyla to identify patterns that can be explored in further studies. To assess whether known ecological trends could be supported by our data, we compared OTU richness in managed and un‐managed sites and OTU richness after a fire at one of our sites. Mixed model regressions with plot and site as random effects were used. All taxa and then subsets of phyla were used to visualise this (the subsets were based on higher abundance phyla to allow for meaningful analysis). NMDS plots were used to visualise community differences by site biome using Jaccard's index. Jaccard's index was used to account for presence and absence since differences in size (physical size of organism, number of gene copies, and potential genome length) of taxonomic groups may skew read counts in our study. We encourage future studies to explore statistical methods to incorporate relative taxon prevalence. PERMANOVA in vegan was used to test for statistical differences (including site and plot as factors), and pairwise comparisons (using FDR corrections) were computed in the ecole package using the function: pairwise adonis.

**TABLE 2:** Correlation values of taxonomic group richness by characteristics of the soils where the sample was collected.

	All	Fungi	Streptophyta	Metazoa	Nematoda	Arthropoda	Annelida
Elevation	0.05	0.05***	−0.02	−0.07	−0.01	−0.07	−0.11*
Latitude	0.30***	0.30***	0.25***	0.12*	0.13*	0.08	0.09
Soil Temp	−0.29***	−0.29***	−0.17	−0.12*	−0.15**	−0.09	−0.03
Soil Moisture	0.08*	0.07	−0.03	0.22***	0.20***	0.14**	0.24***
Soil pH (water)	−0.31***	−0.31***	0.01	−0.33***	−0.21***	−0.28***	−0.24***
% N	0.16***	0.16**	0	0.34***	0.19***	0.29***	0.26***
% Organic C	0.22***	0.22***	0	0.39***	0.21***	0.36***	0.23***
Ammonium	0.01	0.01	−0.06	0.07	−0.05	0.1	0.04
Nitrate	−0.12	−0.12*	−0.01	−0.02	−0.1	0.02	−0.08

*Note:* Significance level is indicated by *< 0.05, **< 0.01, ***< 0.001. Significance is determined by mixed models including plot and site as random effects. Negative values are coloured red and positive values are coloured in blue. Colours are scaled by magnitude of correlation.

## 3 Results

### 3.1 Eukaryote Sequences in NEON Shotgun Metagenome Datasets

We recovered ~1.36 × 10^6^ reads aligning to eukaryotic 18S rRNA references across 45 sites, 6 years, and 1305 samples (mean ~22,000 reads per site‐year combination, ~800 per sample) with an average read length of 138 bp, which is sufficient to identify taxa to at least families in eukaryotes (Wu et al. 2015). Our filtering was stringent and excluded an average of 75% of extracted 18S sequences from the final analysis (Table S1). Our reads corresponded to 5183 genera belonging to 35 phyla, including all common soil animal, many protist, and prominent fungal phyla (e.g., Arthropoda, Nematoda, Rotifera, Tardigrada, Ciliophora, Cercozoa, Tubulinea, Evosea, Chlorophyta, Stramenopiles, Apicomplexa, Euglenozoa, and Ascomycota), a diversity of genera similar to that found in similar studies (Delgado‐Baquerizo et al. 2018; Aslani et al. 2022; Vasar et al. 2022). Eukdetect recovered 35 genera from a subset of 280 samples from 36 sites, with 34 (97%) assigned to kingdom Fungi. In comparison, only ~34% of genera (1759 of 5183) recovered with this study's barrnap hmm profile approach were Fungi (Table S2).

### 3.2 Distribution of Major Taxonomic Groups in the NEON Data Set

**FIGURE 3:**
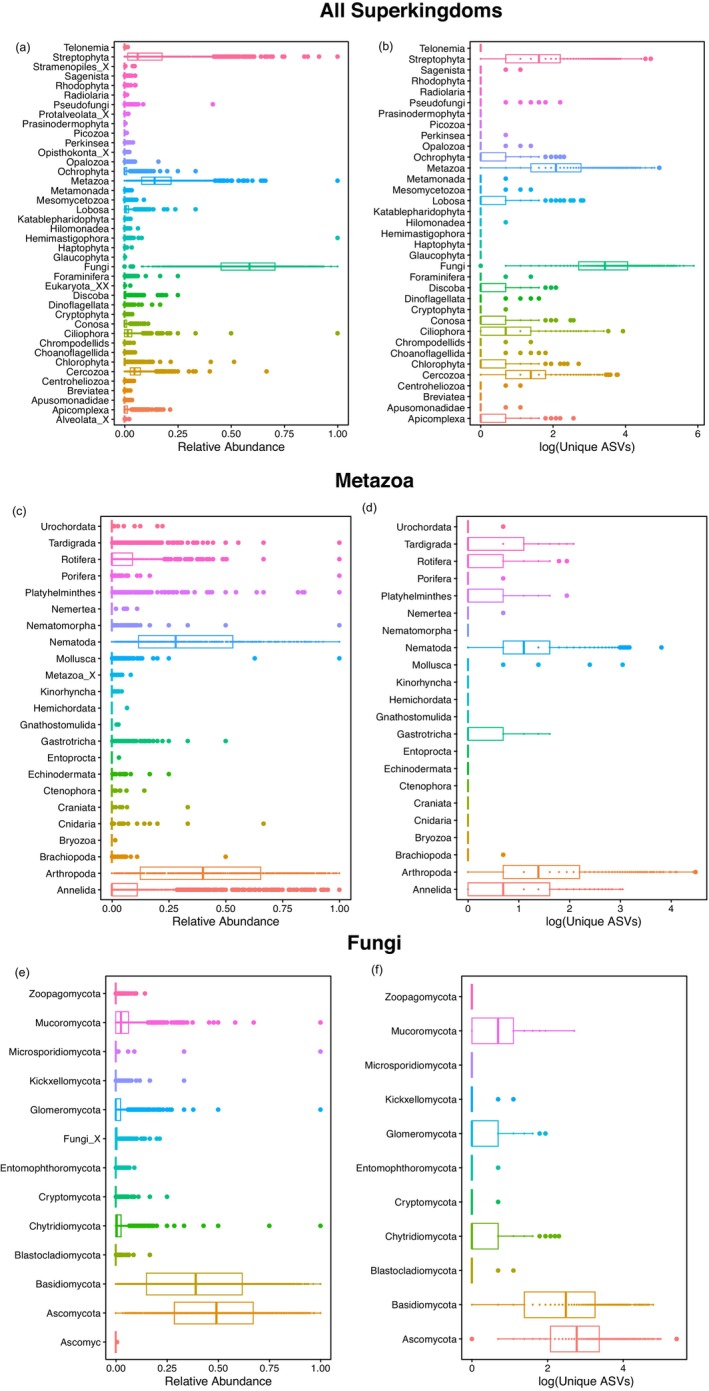
Boxplots of the relative abundances of all superkingdoms (a), metazoa (c), and fungi (e). Boxplots of log(unique OTUs) of all superkingdoms (b), metazoa (d), and fungi (f). Boxplots represent the median with the colored line and whiskers with the 5th and 95th percentiles. All samples are shown.

Our pipeline recovered 35 kingdoms including Fungi, Rhizaria, Metazoa, and Streptophyta (Figure 3). Fungi were the most diverse with 1734 OTUs, then Metazoa with 1458 OTUs, Streptophyta with 801 OTUs, and Cercozoa with 132 OTUs (Figure 3a,b). Within Fungi, Ascomycota was the most diverse phylum with 906 OTUs, then Basidiomycota with 475 OTUs, and Mucoromycota with 33 OTUs. Within Metazoa, Arthropoda was the most diverse phylum with 846 unique OTUs, then Nematoda with 242 OTUs, and Annelida with 127 OTUs.

### 3.3 Trends in the Eukaryotic Community Data

Our study also measured correlations between sample taxonomic richness (number of unique OTUs) and sample characteristics (Table 2). Total eukaryote richness was positively correlated with latitude, soil moisture, percent nitrogen, and percent organic carbon, and negatively correlated with soil temperature and soil pH (Table 2). Across all higher taxonomic levels, these trends remained the same, except for Streptophyta, where only latitude was significantly and positively correlated. For the other lower taxonomic groups, the trends followed that of total eukaryote richness, except for latitude and soil temperature for Arthropoda and Annelida (Table 2). Contrary to patterns typically observed for aboveground biodiversity, where latitude (Hillebrand 2004) negatively correlated with richness, the richness of several taxa (Fungi, Metazoa, Streptophyta, and Nematoda) was positively related to latitude, albeit weakly.

The richness of all taxa except fungi was positively correlated with soil moisture, while fungal richness was unrelated, perhaps due to their greater drought tolerance than most other soil biota (Cosme 2023). Given the important role that organic matter plays at the base of the soil food web, it was unsurprising that the richness of all heterotrophic taxa was positively related to soil organic C content, while Streptophyta (autotrophs) richness was unrelated to organic C content or N content. Inorganic N (nitrate and ammonium) availability was generally unrelated to taxon richness.

### 3.4 Do the Patterns From This Data Match Established Ecological Trends?

**FIGURE 4:**
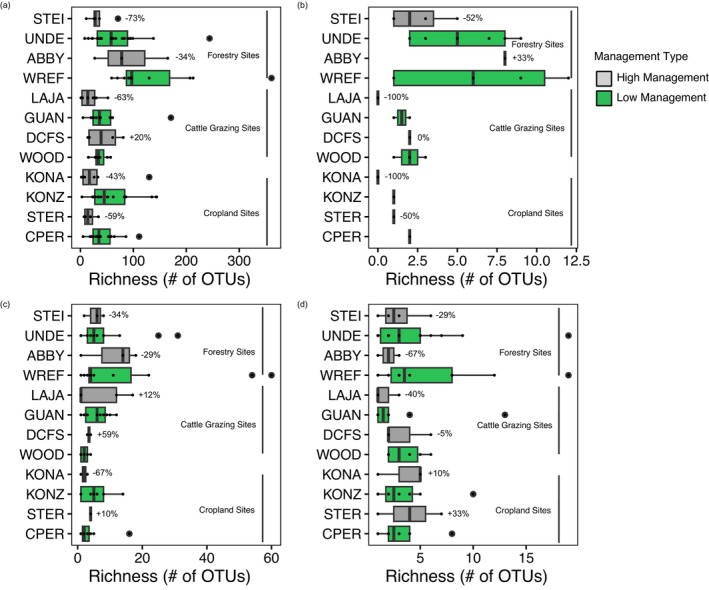
Richness (# of OTUs) by paired management sites. Box plots represent the median with the black line and whiskers with the 5th and 95th percentiles. High management sites are shown in grey and low management sites are shown in green. STEI, UNDE, ABBY, and WREF are forestry sites; LAJA, GUAN, DCFS, and WOOD are cattle grazing sites; and KONA, KONZ, STER, and CPER are cropland sites. Percent reduction of the mean from paired low management and high management sites is shown to the right of the high management sites. (a) Depicts all taxa, (b) depicts the phylum Annelida, (c) depicts the phylum Arthropoda, and (d) depicts the phylum Nematoda.

To test our approach's ability to capture ecologically relevant soil biodiversity trends using NEON datasets, we compared our results to generally accepted ecological patterns. First, we compared the number of unique OTUs at sets of nearby paired sites with higher and lower management intensities (two pairs each for forestry, cattle grazing, and cropland management). Site pairs with low‐high management were UNDE‐STEI and WREF‐ABBY (forestry), GUAN‐LAJA and WOOD‐DCFS (cattle grazing), and KONZ‐KONA and CPER‐STER (cropland). We compared among all taxa, Annelida, Arthropoda, and Nematoda. For all taxa, we found that sites with lower management intensities had higher richness (# of unique OTUs) compared to higher management intensity sites (Figure 4a; *p* = 0.03), with typically 30 fewer genus‐level OTUs (47% reduction in mean richness) at sites with higher management intensities. Unlike most pairs, there was little difference in the mean richness of the WOOD and DCFS sites, which might result from the relatively low grazing intensity at DCFS (https://www.neonscience.org/field‐sites/dcfs). Mean richness was also similar at WREF and ABBY, which is more surprising given that ABBY was logged and re‐planted with Douglas fir around 2005 (although it did retain small patches of mature trees), whereas WREF is old‐growth forest. Among the more intensively managed sites, croplands and grazing lands had the lowest richness (45 and 36 OTUs, respectively), while sites used for forestry had the highest richness (83 OTUs), possibly reflecting differences in management intensity as well as geographic and ecoclimatic differences. For the phylum Annelida, we found the same trend as for all taxa with a lower mean richness in higher management intensity sites (*p* = 0.007; Figure 4b). On average, there were 3.5 fewer OTUs (a 78% reduction in mean richness). There was also lower richness in cropland (0.5 OTUs) and grazing‐land sites (0.75 OTUs) compared to forestry sites (5 OTUs). For phyla Arthropoda (Figure 4c) and Nematoda (Figure 4d), we found no significant differences in management (*p* = 0.6 and 0.4, respectively).

**FIGURE 5:**
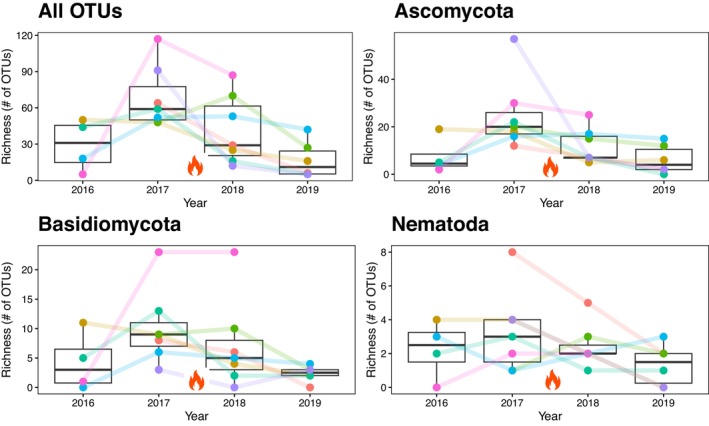
Richness (# of OTUs) of four CLBJ plots by year that were burned in between sampling between 2017 and 2018. Boxplots represent the median with the black line and whiskers with the 5th and 95th percentiles. All points are shown in colours. Lines represent plot changes over time. Analyses were conducted on all taxa and subsets of phyla: Ascomycota, Basidiomycota, and Nematoda.

The second test was whether fire had an impact on soil eukaryotes. In one site—CLBJ from north‐central Texas—there was a fire in several plots between 2017 and 2018. Richness was highest for all taxa before the fire (in 2017) then gradually decreased from 2018 to 2019 (Figure 5). Year was a significant parameter in our mixed model (*p* = 0.007) and richness was significantly lower in 2019 than in 2017 (*p* = 0.008). We also tested these patterns with only the phyla Ascomycota, Basidiomycota, and Nematoda and found the same pattern as for all taxa grouped, with significantly lower richness in 2019 than in 2017 for Ascomycota (*p* = 0.023) and year as significant in our mixed model (*p* = 0.01). There were similar trends for Basidiomycota and Nematoda richness, but they were not significant (*p* = 0.09 and *p* = 0.16, respectively).

**FIGURE 6:**
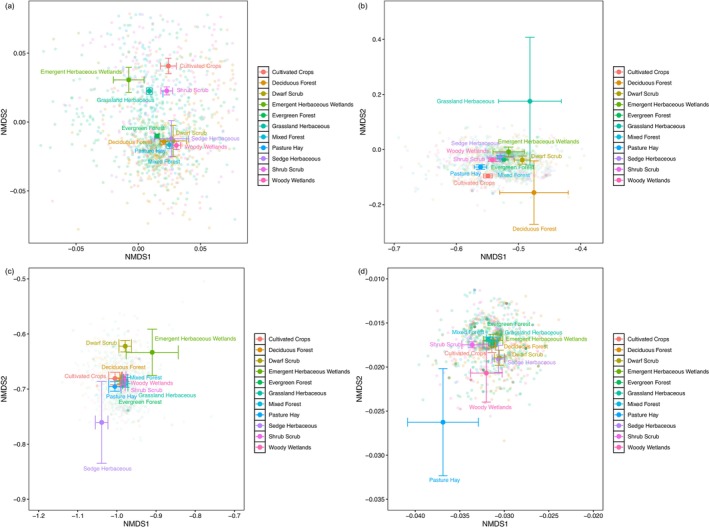
NMDS plot of the 11 different biomes in our study. Transparent points are shown in the background. The mean of all the points is shown with standard error bars. Names correspond to the color of the points. (a) Depicts all taxa, (b) depicts the phylum Ascomycota, (c) depicts the phylum Arthropoda, and (d) depicts the phylum Nematoda.

The third pattern we assessed was whether distinct biomes hosted unique eukaryotic communities. We used PERMANOVAs and NMDS using Jaccard's index (to account for taxon presence/absence only) to quantify and visualize these trends. For all taxa, most biomes possessed a unique eukaryote community (Figure 6a; PERMANOVA *p*‐value = 0.001; Table S3). However, as betadisper was also significant (*p* < 0.001), the diversity within sites and biomes could confound our results. We also measured bray‐curtis within site (Figure S3) and standard deviation by site (Figure S4) and found high dissimilarity among sites and a wide standard deviation. When we ran pairwise comparisons, we found that most biomes were significantly different from one another (*p* < 0.05), except for shrub scrub vs. emergent herbaceous wetlands, pasture hay vs. emergent herbaceous wetlands, and deciduous forest vs. woody wetlands, which were not significantly different from one another when looking at multiple comparison adjusted (FDR) *p*‐values (Table S3). Exploring phylum‐level differences revealed that Ascomyota (Figure 6b) had a significant PERMANOVA (*p* = 0.001), but also a significant betadisper (*p* < 0.001), which potentially confounds our conclusions as previously mentioned for the all taxa group. For pairwise comparisons, we found that all comparisons were significant except: nixed forest vs. woody wetlands, dwarf scrub vs. sedge herbaceous, emergent herbaceous wetlands vs. sedge herbaceous, and deciduous forest vs. woody wetlands (Table S4). For the phylum Arthropoda (Figure 6c), we found a significant PERMANOVA (*p* = 0.001), but also a significant betadisper (*p* < 0.001). For pairwise comparisons, there were 32 significant comparisons and 23 non‐significant comparisons (see Table S5 for further details). The phylum Nematoda (Figure 6d) likewise had a significant PERMANOVA (*p* = 0.001) but also a significant betadisper (*p* < 0.001). For pairwise comparisons, there were 19 significant comparisons and 36 non‐significant comparisons (see Table S6 for more details).

**FIGURE 7:**
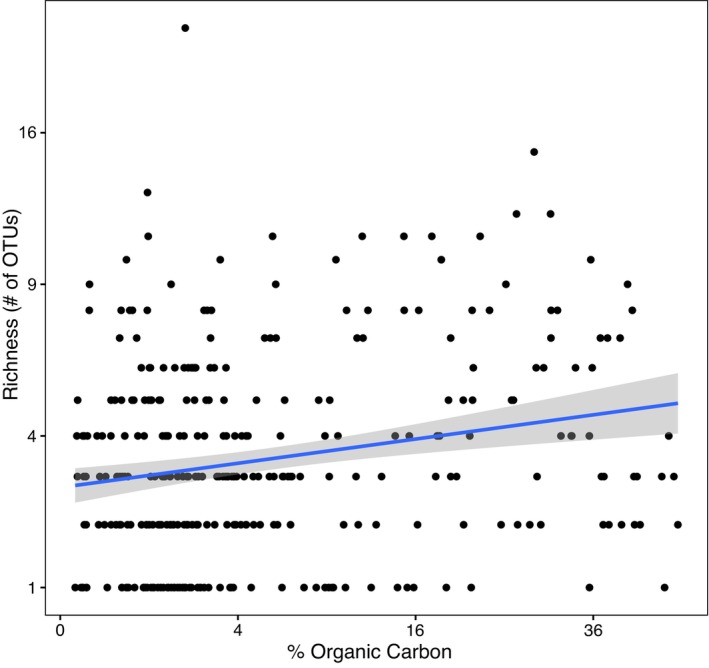
Richness (# of OTUs) over organic % C of nematoda only. Both richness and % Organic C were square root transformed to meet assumptions of a linear regression. Back‐transformed counts are shown in the graph. The linear regression line is shown.

Finally, we attempted to find trends between richness (number of unique OTUs) and site characteristics. Here, we explore one such trend, as an exhaustive exploration of these trends is beyond the scope of our study. Since nematodes have been significantly correlated with organic carbon (Martin and Sprunger 2021), we checked for that relationship in our data. We plotted Nematoda richness (# of OTUs) against organic carbon (Figure 7) and found a positive relationship that was significant when taking the square root of both organic carbon and richness and using site and plot as nested random effects (*p* = 0.003).

## 4 Discussion

### 4.1 Custom Pipeline Allows for Quick and Easy Processing of the Data

The NEON metagenomics data was produced for researchers to evaluate soil microbial communities across the US (Werbin et al. 2021). We repurposed these data to evaluate eukaryotic soil communities and encourage others to further explore this valuable dataset for deeper insights into trends within soil eukaryotic communities and soil characteristics.

Our approach recovered greater eukaryotic diversity than did a recently developed tool, Eukdetect, likely due to differences in strategy and database. First, Eukdetect searches query metagenomes for all markers corresponding to eukaryotes in its reference dataset and only calls a taxon as present if more than a certain percentage of the query aligns. Though a robust approach, this can produce false negatives in high‐complexity environments like soil, as eukaryote sequences are much rarer in soil shotgun metagenomes. Ribosomal sequences are relatively more abundant and thus can serve as a good target for taxonomic assessment when sequencing is shallow. As NEON metagenomes were produced using protocols standard for prokaryotes (i.e., 0.25 g soil extracted, 2 × 150 read inserts, and standard sequencing; NEON 2022a), eukaryote taxa were more likely to be missed due to insufficient DNA extraction volume and sequencing depth, or misidentified due to insufficient read lengths. Though targeting the 18S gene alone is also limited (e.g., low taxonomic resolution in eukaryotes and higher misidentification rate due to shorter insert lengths), it has the potential to be more sensitive in datasets with low average coverage due to higher source complexity.

### 4.2 Data Validation and Example Use Cases

A traditional validation of the metagenomics‐based soil biodiversity data that we generated might involve comparisons to data collected via traditional methods (e.g., microscopy and visual identification). However, given the geographic and taxonomic scope of the dataset, such an approach is not feasible, and existing datasets are neither taxonomically comprehensive enough nor span the sites encompassed in the NEON datasets. Instead, we validated the dataset by exploring it for expected patterns of diversity and responses to disturbance.

To test for ecologically significant trends, we ran correlations with soil characteristics and taxonomic richness. We computed these correlations for all taxa and then also subsets of taxa (Table 2). For all taxa, there were several highly significant correlations found; however, many of these correlations were rather weak. This suggests that environmental drivers of eukaryotic richness are group specific, though broadly latitude, soil temperature, soil moisture, soil pH, and carbon and nitrogen influence taxonomic distribution at the domain level, albeit weakly. Previous studies have shown that mean annual precipitation predicted soil eukaryote richness most strongly, and our study found that soil moisture was indeed significantly and weakly correlated with most taxon richness (Aslani et al. 2022). Soil pH was likewise significant and weakly correlated, though more analyses are needed to confirm this trend. Soil pH has been shown to be highly correlated with soil microbial communities (Fierer and Jackson 2006; Wang et al. 2019; Aslani et al. 2022) and may be correlated with eukaryotic communities as well (Köninger et al. 2023), though the differing methodologies across these studies complicate direct comparison with our own findings. Interestingly, we found a consistent positive relationship with elevation for all taxa as a group as well as Fungi, Streptophyta, Metazoa, and Nematoda individually, contradicting the findings of current aboveground studies (Hillebrand 2004), though belowground studies showed no relationship (Fungi: Dennis et al. 2012; microscopic eukaryotes: Shen et al. 2014) or a negative relationship (protists; Huang et al. 2023). Our correlations were rather weak; therefore, it is possible that either our large sample size created false positives in our statistical models, colder biomes (higher latitude) may preserve DNA better than warmer biomes (lower latitudes) (Kjær et al. 2022), or niche differentiation may allow soil eukaryotes to adapt to colder biomes (Wang et al. 2021). Overall, we recovered patterns consistent with previously established ecological trends, with the exception of latitude, which warrants further investigation.

We also evaluated OTU richness at site pairs with low and high management intensity. We looked at six paired sites where one had lower management intensity and the other had higher management intensity. High management intensity in our case referred to forest management, cattle grazing, and croplands whereas low management referred to minimally managed forests and grasslands. We expected higher richness in low management sites as those should have experienced less human disturbance and pollution. In the paired sites, low management sites generally had higher richness than high management sites (Figure 4a), which is consistent with studies showing decreased biodiversity at managed sites (Paillet et al. 2010; Qu et al. 2024). However, phylum‐level responses to management intensity were varied. Phylum Annelida experienced decreased richness in high management sites compared to low management (Figure 4b), while Arthropoda and Nematoda showed no significant difference between management intensities, indicating that these trends may be phylum specific. We encourage future studies to analyse trends in other phyla not covered in this study.

At the CLBJ site (north‐central Texas) there was a fire in several of the plots between 2017 and 2018 (Figure 5), with a decrease in total richness after the fire (from 2017 to 2019). This finding parallels other studies that have shown decreases in soil eukaryotes due to fire (Moretti et al. 2006; Certini et al. 2021). When we evaluated phylum‐level differences, we found again that the responses were phylum specific. For example, Ascomycota decreased from 2017 to 2019 (Figure 5b), but neither Basidiomycota (Figure 5c) nor Nematoda (Figure 5d) did. Since Ascomycota is a highly abundant fungi and fungi were the most abundant kingdom in our study, Ascomycota alone could be driving the recovered trend for all taxa as a group. We did not assess other natural disasters such as, hurricanes, climate change, or temperature rise and their impact on soil eukaryote communities, but future studies should use the NEON data to evaluate the effects of these and other natural disasters.

Finally, we examined how community composition corresponded to NEON‐assigned biomes. To test whether communities from different biomes were distinct, we used beta diversity measurements. Most of the biomes had unique communities (significant differences measured by PERMANOVAs) except for a few biomes (Figure 6a; Table S3). With this large of a dataset, it is not surprising that communities differed significantly by biome. Such patterns, while deserving further ecological investigation, help validate our pipeline's utility and are consistent with previous work showing unique eukaryotic community composition across biomes (Köninger et al. 2023). Further, when delving deeper into phylum‐level differences, we found that for the phylum Ascomycota (Figure 6b; Table S4), several of the forest biomes (Mixed Forest, Woody Wetlands, and Deciduous Forest) did not differ significantly, but all other biomes were significantly different. The clustering of forest biomes suggests that Ascomycota community composition in forest soils is driven by factors which (1) are relatively constant across latitudinal and altitudinal gradients and (2) differ from those driving plant communities. For Arthropoda (Figure 6c; Table S5), most biomes clustered except for Sedge Herbaceous, Dwarf Scrub, and Emergent Herbaceous Wetlands, suggesting that Arthropoda communities were more similar across biomes than other phyla. For Nematoda (Figure 5d; Table S6), most biomes were not significantly different from one another except for Shrub Scrub, indicating again that Nematoda composition may not vary much between biomes, except in a few distinct biomes.

Despite the limited depth of our reads and strength of our analyses, we recovered well‐established ecological trends, except for a positive relationship of elevation with OTU richness. We are confident that our findings indicate that NEON shotgun metagenomes can be used to explore soil eukaryote diversity and distribution. The NEON datasets are large, well documented, and well supported, and are thus ripe for broader exploration of eukaryotic trends than we have shown here. For example, we only evaluated prominent opisthokonts at the phylum level (Fungi and Metazoa), but many important eukaryote phyla are outside these lineages (Geisen et al. 2018). Additionally, studies could analyse trends at higher taxonomic levels (reliably to family), explore the relationship of eukaryotic richness with latitude and other environmental factors we didn't discuss, as well as evaluate further phylum level differences or search for more trends present in the literature (e.g., effects of drought, deluge, hurricanes, and temperature). Moreover, future studies could compare the richness obtained from multiple approaches and databases (e.g., Metaxa2 with SILVA and Eukdetect). Finally, improving tool sensitivity and database breadth will allow for analyses at higher taxonomic levels (e.g., family and genus) and more robust statistical tests of underexplored datasets like NEON shotgun metagenomes.

## Author Contributions

Contributed to conception and design: A.T., B.A., E.A., A.L.C.F., D.H.W. Contributed to pipeline design and development: A.T. Contributed to analysis of data: L.V., A.T. Contributed to interpretation of data: L.V., A.T., B.A., E.A., A.L.C.F., D.H.W. Drafted and/or revised the article: L.V., A.T., B.A., E.A., A.L.C.F., D.H.W. Approved the submitted version for publication: L.V., A.T., B.A., E.A., A.L.C.F., D.H.W.

## Conflicts of Interest

The authors declare no conflicts of interest.

## Supporting information


**Figure S1:** Rarefication curve of all samples.
**Figure S2:** Species accumulation curve.
**Figure S3:** Bray curtis distance by site.
**Figure S4:** Richness of OTUs and standard deviation by site.
**Table S1:** Sequence summary statistics.
**Table S2:** Eukdetect summary.
**Table S3:** Multiple test adjusted *p*‐values of all comparisons of biome beta diversity for all OTUs using FDR corrections.
**Table S4:** Multiple test adjusted *p*‐values of all comparisons of biome beta diversity for Ascomycota OTUs using FDR corrections.
**Table S5:** Multiple test adjusted *p*‐values of all comparisons of biome beta diversity for Arthropoda OTUs using FDR corrections.
**Table S6:** Multiple test adjusted *p*‐values of all comparisons of biome beta diversity for Nematoda OTUs using FDR corrections.

## Data Availability

The pipeline is publicly available on GitHub and Zenodo at: https://github.com/Andy‐Thmpsn/metagenomics‐18S‐extraction‐pipeline and DOI: https://doi.org/10.5281/zenodo.17162900. All analysed data and R code is available on GitHub and Zenodo at: https://github.com/Leena312/metagenomics‐18S‐R‐code‐and‐files or DOI: https://doi.org/10.5281/zenodo.17161624. All raw data is available at the NEON metagenomes portal. https://www.neonscience.org/resources/learning‐hub/tutorials/neon‐data‐metagenomics.
