# Supplementary material for: Characterising Soil Eukaryotic Diversity From NEON Metagenomics Datasets
Source: Mol Ecol Resour. 2025 Oct 22;26(1):e70062. doi: 10.1111/1755-0998.70062 (PMC12627910; doi:10.1111/1755-0998.70062)
Supplement: Supplementary file 1 — Figure S1: Rarefication curve of all samples. Figure S2: Species accumulation curve. Figure S3: Bray curtis distance by site. Figure S4: Richness of OTUs and standard deviation by site. Table S1: Sequence summary statistics. Table S2: Eukdetect summary. Table S3: Multiple test adjusted p‐values of all comparisons of biome beta diversity for all OTUs using FDR corrections. Table S4: Multiple test adjusted p‐values of all comparisons of biome beta diversity for Ascomycota OTUs using FDR corrections. Table S5: Multiple test adjusted p‐values of all comparisons of biome beta diversity for Arthropoda OTUs using FDR corrections. Table S6: Multiple test adjusted p‐values of all comparisons of biome beta diversity for Nematoda OTUs using FDR corrections. [file MEN-26-e70062-s001.zip › men70062-sup-0001-TableS1-S6-FigureS1-S4@Supplementary Information.docx]

**Supplementary Information**

**Tables S1 and S2 are available as a separate Excel filel: “Supplementary Tables S1 and S2”**

**Table S3.**

Multiple test adjusted p-values of all comparisons of biome beta diversity for all OTUs using FDR corrections.

| pairs | p.adj |
| --- | --- |
| deciduousForest vs mixedForest | 0.0023913 |
| deciduousForest vs evergreenForest | 0.00127907 |
| deciduousForest vs grasslandHerbaceous | 0.00127907 |
| deciduousForest vs cultivatedCrops | 0.00127907 |
| deciduousForest vs pastureHay | 0.00127907 |
| deciduousForest vs woodyWetlands | 0.111 |
| deciduousForest vs shrubScrub | 0.00127907 |
| deciduousForest vs emergentHerbaceousWetlands | 0.00127907 |
| deciduousForest vs dwarfScrub | 0.00127907 |
| deciduousForest vs sedgeHerbaceous | 0.00127907 |
| mixedForest vs evergreenForest | 0.0023913 |
| mixedForest vs grasslandHerbaceous | 0.00127907 |
| mixedForest vs cultivatedCrops | 0.00127907 |
| mixedForest vs pastureHay | 0.00127907 |
| mixedForest vs woodyWetlands | 0.00785714 |
| mixedForest vs shrubScrub | 0.00127907 |
| mixedForest vs emergentHerbaceousWetlands | 0.00127907 |
| mixedForest vs dwarfScrub | 0.00127907 |
| mixedForest vs sedgeHerbaceous | 0.00127907 |
| evergreenForest vs grasslandHerbaceous | 0.00127907 |
| evergreenForest vs cultivatedCrops | 0.00127907 |
| evergreenForest vs pastureHay | 0.00127907 |
| evergreenForest vs woodyWetlands | 0.00127907 |
| evergreenForest vs shrubScrub | 0.00127907 |
| evergreenForest vs emergentHerbaceousWetlands | 0.00127907 |
| evergreenForest vs dwarfScrub | 0.00127907 |
| evergreenForest vs sedgeHerbaceous | 0.00127907 |
| grasslandHerbaceous vs cultivatedCrops | 0.00127907 |
| grasslandHerbaceous vs pastureHay | 0.00127907 |
| grasslandHerbaceous vs woodyWetlands | 0.00127907 |
| grasslandHerbaceous vs shrubScrub | 0.00127907 |
| grasslandHerbaceous vs emergentHerbaceousWetlands | 0.01375 |
| grasslandHerbaceous vs dwarfScrub | 0.00127907 |
| grasslandHerbaceous vs sedgeHerbaceous | 0.00127907 |
| cultivatedCrops vs pastureHay | 0.00351064 |
| cultivatedCrops vs woodyWetlands | 0.00127907 |
| cultivatedCrops vs shrubScrub | 0.00127907 |
| cultivatedCrops vs emergentHerbaceousWetlands | 0.0023913 |
| cultivatedCrops vs dwarfScrub | 0.00127907 |
| cultivatedCrops vs sedgeHerbaceous | 0.00127907 |
| pastureHay vs woodyWetlands | 0.00127907 |
| pastureHay vs shrubScrub | 0.00127907 |
| pastureHay vs emergentHerbaceousWetlands | 0.10898148 |
| pastureHay vs dwarfScrub | 0.00127907 |
| pastureHay vs sedgeHerbaceous | 0.00127907 |
| woodyWetlands vs shrubScrub | 0.00127907 |
| woodyWetlands vs emergentHerbaceousWetlands | 0.00127907 |
| woodyWetlands vs dwarfScrub | 0.00127907 |
| woodyWetlands vs sedgeHerbaceous | 0.00127907 |
| shrubScrub vs emergentHerbaceousWetlands | 0.0809434 |
| shrubScrub vs dwarfScrub | 0.00127907 |
| shrubScrub vs sedgeHerbaceous | 0.00127907 |
| emergentHerbaceousWetlands vs dwarfScrub | 0.00458333 |
| emergentHerbaceousWetlands vs sedgeHerbaceous | 0.01078431 |
| dwarfScrub vs sedgeHerbaceous | 0.0088 |

**Table S4.**

Multiple test adjusted p-values of all comparisons of biome beta diversity for Ascomycota OTUs using FDR corrections.

| pairs | p.adj |
| --- | --- |
| deciduousForest vs mixedForest | 0.00895349 |
| deciduousForest vs evergreenForest | 0.00157143 |
| deciduousForest vs grasslandHerbaceous | 0.00157143 |
| deciduousForest vs cultivatedCrops | 0.00157143 |
| deciduousForest vs pastureHay | 0.00157143 |
| deciduousForest vs woodyWetlands | 0.12692308 |
| deciduousForest vs shrubScrub | 0.00157143 |
| deciduousForest vs emergentHerbaceousWetlands | 0.0055 |
| deciduousForest vs dwarfScrub | 0.00157143 |
| deciduousForest vs sedgeHerbaceous | 0.00157143 |
| mixedForest vs evergreenForest | 0.04637255 |
| mixedForest vs grasslandHerbaceous | 0.00157143 |
| mixedForest vs cultivatedCrops | 0.00157143 |
| mixedForest vs pastureHay | 0.00157143 |
| mixedForest vs woodyWetlands | 0.224 |
| mixedForest vs shrubScrub | 0.00157143 |
| mixedForest vs emergentHerbaceousWetlands | 0.00977778 |
| mixedForest vs dwarfScrub | 0.00157143 |
| mixedForest vs sedgeHerbaceous | 0.00157143 |
| evergreenForest vs grasslandHerbaceous | 0.00157143 |
| evergreenForest vs cultivatedCrops | 0.00157143 |
| evergreenForest vs pastureHay | 0.00157143 |
| evergreenForest vs woodyWetlands | 0.00157143 |
| evergreenForest vs shrubScrub | 0.00157143 |
| evergreenForest vs emergentHerbaceousWetlands | 0.00297297 |
| evergreenForest vs dwarfScrub | 0.00157143 |
| evergreenForest vs sedgeHerbaceous | 0.00157143 |
| grasslandHerbaceous vs cultivatedCrops | 0.00157143 |
| grasslandHerbaceous vs pastureHay | 0.0055 |
| grasslandHerbaceous vs woodyWetlands | 0.00157143 |
| grasslandHerbaceous vs shrubScrub | 0.00157143 |
| grasslandHerbaceous vs emergentHerbaceousWetlands | 0.0055 |
| grasslandHerbaceous vs dwarfScrub | 0.00157143 |
| grasslandHerbaceous vs sedgeHerbaceous | 0.00157143 |
| cultivatedCrops vs pastureHay | 0.00157143 |
| cultivatedCrops vs woodyWetlands | 0.00157143 |
| cultivatedCrops vs shrubScrub | 0.00157143 |
| cultivatedCrops vs emergentHerbaceousWetlands | 0.00157143 |
| cultivatedCrops vs dwarfScrub | 0.00157143 |
| cultivatedCrops vs sedgeHerbaceous | 0.00157143 |
| pastureHay vs woodyWetlands | 0.01195652 |
| pastureHay vs shrubScrub | 0.00157143 |
| pastureHay vs emergentHerbaceousWetlands | 0.02581633 |
| pastureHay vs dwarfScrub | 0.00297297 |
| pastureHay vs sedgeHerbaceous | 0.00670732 |
| woodyWetlands vs shrubScrub | 0.00157143 |
| woodyWetlands vs emergentHerbaceousWetlands | 0.00785714 |
| woodyWetlands vs dwarfScrub | 0.00157143 |
| woodyWetlands vs sedgeHerbaceous | 0.00157143 |
| shrubScrub vs emergentHerbaceousWetlands | 0.01404255 |
| shrubScrub vs dwarfScrub | 0.00977778 |
| shrubScrub vs sedgeHerbaceous | 0.0240625 |
| emergentHerbaceousWetlands vs dwarfScrub | 0.0418 |
| emergentHerbaceousWetlands vs sedgeHerbaceous | 0.14424528 |
| dwarfScrub vs sedgeHerbaceous | 0.14462963 |

**Table S5.**

Multiple test adjusted p-values of all comparisons of biome beta diversity for Arthropoda OTUs using FDR corrections.

| pairs | p.adj |
| --- | --- |
| deciduousForest vs mixedForest | 0.11987179 |
| deciduousForest vs evergreenForest | 0.00982143 |
| deciduousForest vs cultivatedCrops | 0.0034375 |
| deciduousForest vs pastureHay | 0.06722222 |
| deciduousForest vs woodyWetlands | 0.43676471 |
| deciduousForest vs shrubScrub | 0.0034375 |
| deciduousForest vs grasslandHerbaceous | 0.0034375 |
| deciduousForest vs emergentHerbaceousWetlands | 0.15845238 |
| deciduousForest vs dwarfScrub | 0.0034375 |
| deciduousForest vs sedgeHerbaceous | 0.0034375 |
| mixedForest vs evergreenForest | 0.49601852 |
| mixedForest vs cultivatedCrops | 0.0034375 |
| mixedForest vs pastureHay | 0.06837838 |
| mixedForest vs woodyWetlands | 0.2921875 |
| mixedForest vs shrubScrub | 0.00785714 |
| mixedForest vs grasslandHerbaceous | 0.0088 |
| mixedForest vs emergentHerbaceousWetlands | 0.18875 |
| mixedForest vs dwarfScrub | 0.0034375 |
| mixedForest vs sedgeHerbaceous | 0.0034375 |
| evergreenForest vs cultivatedCrops | 0.0055 |
| evergreenForest vs pastureHay | 0.03193548 |
| evergreenForest vs woodyWetlands | 0.49288462 |
| evergreenForest vs shrubScrub | 0.0034375 |
| evergreenForest vs grasslandHerbaceous | 0.0055 |
| evergreenForest vs emergentHerbaceousWetlands | 0.18875 |
| evergreenForest vs dwarfScrub | 0.0034375 |
| evergreenForest vs sedgeHerbaceous | 0.0034375 |
| cultivatedCrops vs pastureHay | 0.561 |
| cultivatedCrops vs woodyWetlands | 0.00982143 |
| cultivatedCrops vs shrubScrub | 0.149875 |
| cultivatedCrops vs grasslandHerbaceous | 0.06722222 |
| cultivatedCrops vs emergentHerbaceousWetlands | 0.06333333 |
| cultivatedCrops vs dwarfScrub | 0.0034375 |
| cultivatedCrops vs sedgeHerbaceous | 0.0034375 |
| pastureHay vs woodyWetlands | 0.11987179 |
| pastureHay vs shrubScrub | 0.30755102 |
| pastureHay vs grasslandHerbaceous | 0.49396226 |
| pastureHay vs emergentHerbaceousWetlands | 0.15845238 |
| pastureHay vs dwarfScrub | 0.0088 |
| pastureHay vs sedgeHerbaceous | 0.0088 |
| woodyWetlands vs shrubScrub | 0.00982143 |
| woodyWetlands vs grasslandHerbaceous | 0.0165 |
| woodyWetlands vs emergentHerbaceousWetlands | 0.19433333 |
| woodyWetlands vs dwarfScrub | 0.0088 |
| woodyWetlands vs sedgeHerbaceous | 0.0034375 |
| shrubScrub vs grasslandHerbaceous | 0.26212766 |
| shrubScrub vs emergentHerbaceousWetlands | 0.20086957 |
| shrubScrub vs dwarfScrub | 0.0034375 |
| shrubScrub vs sedgeHerbaceous | 0.0055 |
| grasslandHerbaceous vs emergentHerbaceousWetlands | 0.3234 |
| grasslandHerbaceous vs dwarfScrub | 0.0034375 |
| grasslandHerbaceous vs sedgeHerbaceous | 0.0055 |
| emergentHerbaceousWetlands vs dwarfScrub | 0.0165 |
| emergentHerbaceousWetlands vs sedgeHerbaceous | 0.06470588 |
| dwarfScrub vs sedgeHerbaceous | 0.0446875 |

**Table S6.**

Multiple test adjusted p-values of all comparisons of biome beta diversity for Nematoda OTUs using FDR corrections.

| pairs | p.adj |
| --- | --- |
| deciduousForest vs mixedForest | 0.64166667 |
| deciduousForest vs evergreenForest | 0.17769231 |
| deciduousForest vs cultivatedCrops | 0.01466667 |
| deciduousForest vs pastureHay | 0.47807692 |
| deciduousForest vs woodyWetlands | 0.3575 |
| deciduousForest vs shrubScrub | 0.00785714 |
| deciduousForest vs grasslandHerbaceous | 0.00785714 |
| deciduousForest vs emergentHerbaceousWetlands | 0.25265625 |
| deciduousForest vs dwarfScrub | 0.01178571 |
| deciduousForest vs sedgeHerbaceous | 0.93805556 |
| mixedForest vs evergreenForest | 0.971 |
| mixedForest vs cultivatedCrops | 0.02138889 |
| mixedForest vs pastureHay | 0.20035714 |
| mixedForest vs woodyWetlands | 0.19148148 |
| mixedForest vs shrubScrub | 0.01 |
| mixedForest vs grasslandHerbaceous | 0.21241379 |
| mixedForest vs emergentHerbaceousWetlands | 0.45189189 |
| mixedForest vs dwarfScrub | 0.077 |
| mixedForest vs sedgeHerbaceous | 0.8448 |
| evergreenForest vs cultivatedCrops | 0.01178571 |
| evergreenForest vs pastureHay | 0.1562 |
| evergreenForest vs woodyWetlands | 0.00785714 |
| evergreenForest vs shrubScrub | 0.00785714 |
| evergreenForest vs grasslandHerbaceous | 0.00785714 |
| evergreenForest vs emergentHerbaceousWetlands | 0.51243902 |
| evergreenForest vs dwarfScrub | 0.01 |
| evergreenForest vs sedgeHerbaceous | 0.87676471 |
| cultivatedCrops vs pastureHay | 0.71270833 |
| cultivatedCrops vs woodyWetlands | 0.02894737 |
| cultivatedCrops vs shrubScrub | 0.2466129 |
| cultivatedCrops vs grasslandHerbaceous | 0.1562 |
| cultivatedCrops vs emergentHerbaceousWetlands | 0.1562 |
| cultivatedCrops vs dwarfScrub | 0.00785714 |
| cultivatedCrops vs sedgeHerbaceous | 0.57174419 |
| pastureHay vs woodyWetlands | 0.73071429 |
| pastureHay vs shrubScrub | 0.51857143 |
| pastureHay vs grasslandHerbaceous | 0.35042857 |
| pastureHay vs emergentHerbaceousWetlands | 0.21266667 |
| pastureHay vs dwarfScrub | 0.0171875 |
| pastureHay vs sedgeHerbaceous | 0.89163462 |
| woodyWetlands vs shrubScrub | 0.00785714 |
| woodyWetlands vs grasslandHerbaceous | 0.01 |
| woodyWetlands vs emergentHerbaceousWetlands | 0.08380952 |
| woodyWetlands vs dwarfScrub | 0.01 |
| woodyWetlands vs sedgeHerbaceous | 0.935 |
| shrubScrub vs grasslandHerbaceous | 0.1562 |
| shrubScrub vs emergentHerbaceousWetlands | 0.35042857 |
| shrubScrub vs dwarfScrub | 0.01941176 |
| shrubScrub vs sedgeHerbaceous | 0.64166667 |
| grasslandHerbaceous vs emergentHerbaceousWetlands | 0.32166667 |
| grasslandHerbaceous vs dwarfScrub | 0.01178571 |
| grasslandHerbaceous vs sedgeHerbaceous | 0.66358696 |
| emergentHerbaceousWetlands vs dwarfScrub | 0.51243902 |
| emergentHerbaceousWetlands vs sedgeHerbaceous | 0.47473684 |
| dwarfScrub vs sedgeHerbaceous | 0.68925532 |

**Supplementary Figures**

*Figure S1. Rarefication curve of all samples.*

**

*Figure S2. Species accumulation curve.*

**

*Figure S3. Bray curtis distance by site*

**

*Figure S4. Richness of OTUs and standard deviation by site*
